# Validation of the German Version of the Music-Empathizing-Music-Systemizing (MEMS) Inventory (Short Version)

**DOI:** 10.3389/fnbeh.2018.00153

**Published:** 2018-08-08

**Authors:** Alexandra Linnemann, Gunter Kreutz, Mario Gollwitzer, Urs M. Nater

**Affiliations:** ^1^Department of Psychiatry and Psychotherapy, University Medical Center Mainz, Mainz, Germany; ^2^Department of Music, School for Linguistics and Cultural Studies, Carl von Ossietzky Universität Oldenburg, Oldenburg, Germany; ^3^Chair of Social Psychology, Department Psychology, Ludwig-Maximilians-Universität Munich, Munich, Germany; ^4^Clinical Psychology, Department of Psychology, University of Vienna, Vienna, Austria

**Keywords:** cognitive style, emotion regulation, music listening, music preference, personality, use of music

## Abstract

**Background:** Kreutz et al. (2008) developed the Music-Empathizing-Music-Systemizing (ME-MS) Inventory to extend Baron-Cohen's cognitive style theory to the domain of music. We sought to confirm the ME-MS construct in a German sample and to explore these individual differences in relation to music preferences.

**Methods:** The German adaptation of the MEMS Inventory was achieved by forward and backward translation. A total of 1014 participants (532 male, age: 33.79 ± 11.89 years) completed the 18-item short version of the MEMS Inventory online. Confirmatory factor analysis (CFA) was performed and cut-off values were established to identify individuals who could be classified as ME, Balanced, or MS. Statistical analyses were used to examine differences in music preference based on music-related cognitive styles.

**Results:** Confirmatory factor analysis (CFA) confirmed two factors, ME and MS, with sufficiently good fit (CFI = 0.87; GFI = 0.93) and adequate internal consistency (Cronbach's Alpha ME: 0.753, MS: 0.783). Analyses of difference scores allowed for a classification as either ME, Balanced, or MS. ME and MS differed in sociodemographic variables, preferred music genres, preferred reasons for music listening, musical expertise, situations in which music is listened to in daily life, and frequency of music-induced chills.

**Discussion:** The German short version of the MEMS Inventory shows good psychometric properties. Based on the cut-off values, differences in music preference were found. Consequently, ME and MS use music in different ways, and the cognitive style of music listening thus appears to be an important moderator in research on the psychology of music. Future research should identify behavioral and neurophysiological correlates and investigate mechanisms underlying music processing based on these different cognitive styles of music listening.

## Introduction

Music listening is associated with complex patterns of neurophysiological activation, spanning from auditory analysis being associated with activity in the auditory cortex, to more complex and subjective interpretations of musical stimuli being associated with activity in limbic and paralimbic regions in the brain (Koelsch and Siebel, 2005; Koelsch, 2010). Most interestingly, music listening affects activity in areas of the brain that are closely related to the modulation of the immune system, neuroendocrine circuits and other stress-sensitive systems in the body, which might explain how music exerts beneficial effects on health (Koelsch, 2010; Chanda and Levitin, 2013).

In this regard, it is of particular interest to examine variations in neurophysiological patterns activated by music based on factors associated with the music itself (e.g., different patterns of electrocardiogram activity evoked by consonant vs. dissonant music; Sammler et al., 2007), with the situation (e.g., different effects of music listening on the hypothalamic-pituitary adrenal axis as measured by cortisol secretion; Linnemann et al., 2016), and with the person (e.g., sex differences in psychophysiological reactivity to music; Nater et al., 2006). In particular, interindividual differences in the use of music, the experiences associated with music, and its underlying neurophysiological mechanisms are of utmost importance in order to tailor specific music interventions based on interindividual needs and preferences.

Previous research has addressed individual differences in musical experiences, preferences, and other aspects of musical engagement with a specific emphasis on personality (Kemp, 1996; Rentfrow and Gosling, 2003; Langendörfer et al., 2006; Chamorro-Premuzic and Furnham, 2007; Greenberg et al., 2015b). One construct that has only recently attracted attention in terms of explaining individual differences in music-related experiences is the so-called Empathizing-Systemizing (E-S) or cognitive style theory (Kreutz et al., 2008; Greenberg et al., 2015a). The original theory by Baron-Cohen (2003) posits that psychological differences between the sexes can be explained by variations along the dimensions of Empathizing and Systemizing. Although the exact neurobiological basis of E-S remains to be elucidated, research has begun to identify neurophysiological correlates of E-S in order to gain a deeper understanding of how behavior, affect and thoughts vary depending on these cognitive styles (Lai et al., 2012). Lai et al. (2012) identified structural differences in brain anatomy based on E-S in men: Whereas S was associated with increased gray matter volume in prefrontal brain areas, E was associated with larger hypothalamic and ventral basal ganglia regions. Thus, these differences in activation of different brain regions based on E-S give rise to the question of whether Baron-Cohen's E-S theory could also be used to explain other facets of human behavior. Van den Brink et al. (2012) were able to associate differences in language processing with differences in event-related potentials, finding that participants who scored high on empathy reacted with larger N400 effects when socially relevant information was processed.

As musical activities are also often discussed to be social activities, they lend themselves to consideration under the perspective of cognitive style theory (Kreutz et al., 2008). On the one hand, music is often characterized as an emotional and social art, which implies that sharing musical thoughts and ideas alludes to core principles of empathizing. Specifically, mental representations of music must entail a degree of social feelings and inter-individual mind states as expressed in synchronous behaviors, such as clapping, foot tapping, dancing, or group singing. On the other hand, music listening is associated with the activation of implicit knowledge of rules that have a strong affinity to music-theoretical principles (Krumhansl, 1990). In other words, even when disregarding the exceptionally high mental and physiological demands of composing and performing music, the psychological reality of music in the everyday listening of untrained individuals is based on their systemic knowledge of music, which is a key characteristic of so-called systemizing. Greenberg et al. (2015a) analyzed the relationships between E-S traits and music preferences with respect to styles, genres, and sonic attributes in large-scale surveys. The authors found that genre preferences were not uniformly distributed across the two cognitive styles. Empathizing was found to be associated with so-called Mellow genres (including R&B/soul, and soft rock), low-arousing, sad and emotionally deep music, whereas Systemizing was associated with a preference for high-arousing music with some affinity to so-called Intense styles (including punk and heavy metal), and music that conveyed a somewhat more positive emotional tone.

It is of note that empathy has recently received increasing interest. Clarke et al. (2015), for example, attribute empathy with a central role in the context of individual and social effects of music. Their literature review suggests that music-induced emotions vary depending on dispositional empathy, which is associated with more intense music-induced emotions. Furthermore, the authors argue that music has the capacity to promote empathy and thus to beneficially affect social bonding and promote cultural understanding. Most interestingly, they do not conceptualize empathy as a fixed trait, but rather as an “environment-complementary action tendency” (Clarke et al., 2015). Thus, people might differ in their degree of empathy in a given situation based on their disposition and on situational factors. The review by Clarke et al. (2015) stimulated a number of comments on the role of empathy for understanding the effects of music. There appears to be consensus that the link between music and empathy is an important one, which deserves attention from various perspectives (cf. Greenberg et al., 2015b). In this regard, research has recently begun to investigate neurophysiological correlates of empathy in the context of music. For example, Wallmark et al. (2018) were able to show that trait empathy modulates music processing, insofar as empathy was correlated with activity in sensorimotor and cognitive areas of the brain.

However, Nettle (2007) conducted an online survey to study individual differences in art appreciation, and observed sex differences that were not fully accounted for by cognitive styles. In other words, the general E-S theory may not be sufficiently sensitive to individual differences in aesthetic experiences, including music. Kreutz et al. (2008) concluded that in order to explore individual differences in the domain of music, it might be necessary to specify cognitive styles further, thus extending the general cognitive styles E-S (Baron-Cohen et al., 2003, 2005). The authors conducted two online surveys, in which specific items for Music Empathizing (ME) and Music Systemizing (MS) were developed. The resulting MEMS Inventory comprises two scales, one for each trait. The short version of this inventory shows good psychometric properties and its ME-MS domains correlate positively with S-E, thus suggesting that ME-MS is similar to the concept of S-E. In fact, female participants in the survey tended to score higher on ME, whereas males tended to score higher on MS. This association was, in part, modulated by musical expertise. In brief, higher proficiency in self-rated musical ability was associated with greater Music Systemizing. In sum, these findings suggest that ME-MS can add distinct information in order to differentiate individual musical experiences and background (Kreutz et al., 2008).

Evidence on associations between ME-MS and musical experiences remains heterogeneous: Recent studies found that scores for ME and MS were correlated with a scale on absorption in music (Sandstrom and Russo, 2013). However, the results were mixed, as both ME and MS were positively associated with absorption in music. As absorption in music is related to strong emotional experiences with music, one might assume that it is positively correlated with ME (but not with MS). Moreover, there is conflicting evidence on whether ME is associated with the enjoyment of negative emotions in music, since one study confirmed this association (Garrido and Schubert, 2011), while another did not (Garrido and Schubert, 2013).

One reason for such heterogeneous findings might lie in the fact that there are two separate scales, namely one to assess ME and one to assess MS. It might be possible that participants show a high score on both scales and thus count as both ME and MS. It is also plausible, however, that a “balanced type” exists, i.e., capturing those individuals who score equally high or low on ME and MS. Accordingly, it is important not only to regard the ME and the MS scores in isolation, but also to relate them to each other. In the context of the E-S theory, Wakabayashi et al. (2006) introduced a way to relate E and S to each other. Based on the distribution of the difference score E-S, the authors determined cut-off values that allow a distinct classification as either E (comprising Empathizers and extreme Empathizers), Balanced, or S (comprising Systemizers and extreme Systemizers) types while relating scores on the E scale to scores on the S scale, thus resulting in one E-S score instead of two separate scores for E and S.

### Aims

The current study had three central aims. First, a German version of the original ME-MS Inventory should be developed using a back-translation procedure in order to explore the feasibility of the construct in a different language culture. Therefore, we sought statistical confirmation of the factor structure using confirmatory factor analysis (CFA). Our second aim was to identify cut-off values that should allow for distinct classifications of individuals as ME, Balanced, or MS. Third, in accordance with Greenberg et al. (2015a), we expected to find associations between the resulting groups of individuals and their music preferences. Furthermore, we hypothesized that ME use music more regularly for emotional reasons than do MS.

## Materials and methods

### The music-empathizing-music-systemizing (MEMS) inventory

The construction of the MEMS Inventory was based on the short version of the general E-S inventory (Wakabayashi et al., 2006). Originally, the MEMS Inventory comprised 44 items (25 representing ME and 19 representing MS). Kreutz et al. (2008) developed a short version of this questionnaire. We presented our participants with the long version of the questionnaire. However, as the long version was psychometrically inferior to the short version (further details on request), we only used the results concerning items of the short version in this paper. This short version of the MEMS scale consists of 18 items, with nine items covering ME and nine items covering MS, respectively. Four items within each domain are negatively poled. Agreement with each item was recorded on 4-point scales ranging from 1 (strongly disagree) to 4 (strongly agree). The short version of the MEMS Inventory was validated in a sample of *N* = 155 participants (50 male, 105 female, age: 30.06 ± 11.77 years) (Kreutz et al., 2008). Based on Principal Component Analysis (PCA), the two factors ME and MS explained 36.69% of the variance. Cronbach's Alpha was 0.69 for ME and 0.81 for MS, respectively, which can be considered as acceptable for MS, whereas the internal consistency of the ME scale is questionable according to common convention. However, as the rather small sample size (implying a narrow range of variability) can deflate this coefficient, a replication in a more heterogeneous sample is necessary.

The German adaptation of the MEMS scale was produced by forward and backward translation. Thus, initially, one translator whose mother tongue is German and who is fluent in English translated the items into German. In an expert panel, these items were then back-translated. In the case of inconsistencies between the original item and the back-translated item, consensus was reached by discussion. In critical cases, a professional translator (native in English and fluent in German) was consulted. Before finalizing the items, we performed a pre-testing by presenting colleagues with the questionnaire and asking for feedback. Feedback was considered for re-wording of the items, thus resulting in a final version of the questionnaire. An overview of the original items and their translations can be found in Table 1.

**Table 1:** MEMS Inventory: internal consistency, factor loadings, and items.

**Subscale**	**Cronbach's Alpha**	**Factor loading**	**Item number**	**Item (German)**	**Item (English)**
Music Empathizing	0.753	0.620	ME03	Ich glaube, ich kann problemlos erkennen, wie sich ein Interpret beim Musizieren fühlt.	I think that I can easily sense how performers feel while playing music.
		0.580	ME06	Ich kann die Gefühle des Interpreten nie erraten. Alternative translation: Ich errate die Gefühle des Interpreten nie.“	I never guess the emotions of the performer(s).
		0.515	ME08	Musik ist für mich hauptsächlich wichtig, weil sie etwas Persönliches und Berührendes ausdrückt.	Music is important to me mainly because it expresses something personal and touching.
		0.376	ME14	Songtexte haben nie eine persönliche Bedeutung für mich.	I never find the lyrics of a song to be meaningful to me.
		0.627	ME15	Wenn ich Musik höre, denke ich über den emotionalen Zustand des Interpreten oder Komponisten zu der Zeit nach, in der das Stück interpretiert wurde.	When listening to music, I have thoughts about the emotional state of the writer/composer at the time.
		0.409	ME16	Ich habe nicht das Gefühl, in der Lage zu sein, mich mit den Sängern/Komponisten meiner Lieblingsmusik zu identifizieren.	I do not feel I am able to identify with the singers/writers of my favorite music.
		0.657	ME17	Wenn ich Musik höre, habe ich das Gefühl, ich verstehe die Emotionen, die der Komponist oder der Interpret versucht auszudrücken.	I feel when listening to music I can understand the emotions the writer/performer is trying to express.
		0.402	ME18	Ich interessiere mich nicht für das Leben meines Lieblingskünstlers zu der Zeit, als er ein bestimmtes Musikstück/Album produziert hat.	I do not care about the lives of my favorite artists at the times they produced a certain song/album.
		0.390	ME23	Ich habe häufig körperliche Empfindungen, wie z.B. Tränen, Schauer etc., wenn ich bestimmte Musikstücke höre.	I often experience physical sensations such as tears, shivers etc when listening to certain pieces of music.
Music Systemizing	0.783	0.604	MS02	Ich interessiere mich nicht für die Struktur eines Musikstücks.	I am not interested in understanding the structure of a piece of music.
		0.559	MS06	Die Physik und Akustik eines Instruments faszinieren mich nicht.	I am not intrigued about the physics and acoustics of musical instruments.
		0.670	MS07	Ich frage mich häufig, wie die mechanischen Einzelheiten eines Musikinstruments funktionieren.	I often wonder how the mechanics of musical instruments work.
		0.698	MS11	Ich mag es, die verschiedenen Schichten von Instrumenten und Stimmen in einem Stück herauszuhören.	I like hearing the different layers of instruments and voices in a song/piece of music.
		0.500	MS13	Ich finde geschriebene Musikpartituren sehr interessant, und besonders gefällt mir die organisierte Art und Weise, in der Musik angelegt ist.	I find written music scores very interesting and I especially like the organized way that music is laid out.
		0.626	MS15	Ich mag es, wie sich ein Musikstück aus seinen Einzelteilen zu einem Ganzen formt.	I like the way a song comes together from all its different parts.
		0.496	MS17	Bei Konzerten faszinieren mich besonders die Rollen der einzelnen Musiker und das daraus entstehende Zusammenspiel.	At concerts, I like to see the roles of the different band/orchestra members and how it all comes together.
		0.188	MS18	Ich mag es, meine Musiksammlung ordentlich sortiert zu haben (z.B. alphabetisch oder nach Genre).	I like to keep my music collection clearly ordered, e.g., alphabetically or by genre.
		0.613	MS19	Ich interessiere mich gar nicht dafür, wie Musik produziert wird und welche Technologien dem zu Grunde liegen.	I am not at all interested in the production side of music and the technologies involved.

*ME, Music Empathizer, MS, Music Systemizer. All negatively poled items have been recoded prior to confirmatory factor analysis*.

### Music preference questionnaire (MPQ-R)

The revised version of the Music Preference Questionnaire was used (German version available here: https://www.musicandhealthlab.com/publications/). This questionnaire comprises nine items that cover music preferences from a multidimensional perspective, thus capturing not only the preference for certain genres but also engagement with music in daily life, musical experiences, importance of music, and the habitual experience of music-induced chills. The first item assesses the preference for music genres by asking respondents to state their preferences on a 5-point Likert scale ranging from not at all (1) to entirely (5) for eleven music genres (pop, rock, hip hop, Latin, soul/funk, hard rock, New Age, Jazz/Blues, electronica, classical, folk music). Subsequent items ask respondents to state their favorite music band and favorite music genre as well as the average amount of time spent on music listening per day. Furthermore, reasons for music listening are assessed and participants are asked to rate the frequency of ten reasons for music listening (e.g., relaxation, activation, distraction, to reduce aggression, to evoke specific emotions) on a 5-point Likert scale ranging from never (1) to frequently (5). Then, situations and occasions in which music is listened to are assessed (e.g., disco/club, concert, when alone, when in the presence of friends). Subsequently, current and past music making is assessed, with participants being asked to indicate whether they play an instrument, sing in a choir or engage in other musical activities. Next, participants are asked to rate the importance of music listening for their own life on a 5-point Likert scale ranging from not at all (1) to entirely important (5). The final item covers the frequency and intensity of habitual music-induced chills.

### Data collection and participants

Participants were recruited online, as the questionnaire was administered via an internet platform. In order to address a diverse sample with respect to age, educational background, and musical expertise, the URL for the survey was sent to university mailing lists, and was posted on social media outlets and on various forums addressing different population groups, e.g., high school students, trainees, housewives and househusbands, retired people, professional, and lay musicians. Written electronic consent was obtained from each participant. The survey was made publicly accessible for 6 weeks from 12th July 2014 to 1st September 2014. Prior to completing the survey, participants were asked to fill out a general demographic background questionnaire. This study was carried out in accordance with the recommendations of the German Society of Psychology. The protocol was approved by the local Ethics Committee of the University of Marburg. All subjects gave written informed consent in accordance with the Declaration of Helsinki. Participation was voluntary and participants could enter a draw to win a tablet worth 200 €.

**Figure 1:**
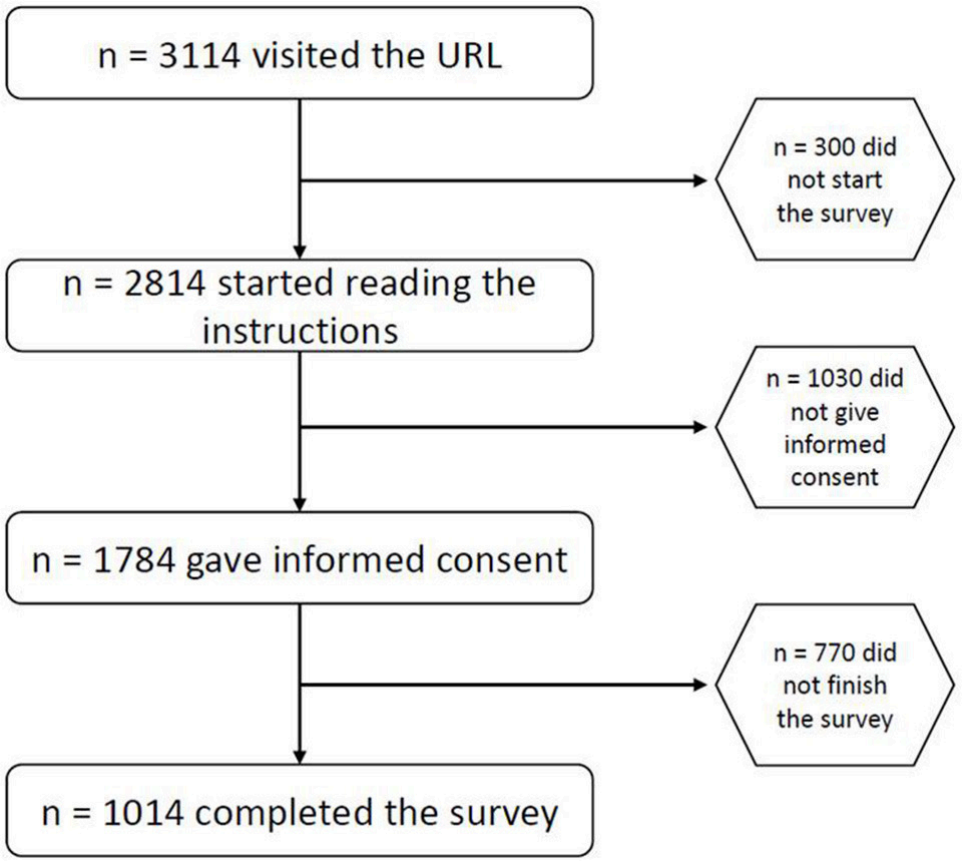
Flow chart diagram.

Inclusion criteria for participation were age ≥18 years and fluency in German. A total of 3,114 persons visited the URL. The completion rate was 32.56%, as a total of *n* = 1,014 participants (532 male, 482 female; mean age: 33.79 ± 11.89, range 18–75) completed the survey in its entirety (see Figure 1). Demographic characteristics of the sample can be found in Table 2.

**Table 2:** Sociodemographic characteristics of the total sample and separately for each cognitive style.

	**Total sample**	***ME***	***B***	***MS***	***p***
Gender					< 0.001
Male	532 (52.5%)	30	367	135	
Female	482 (47.5%)	113	340	29	
Age-mean (SD)	33.79 (11.89)	30 (10)	34 (12)	37 (12)	< 0.001
Marital Status					0.001
Unmarried	718 (70.8%)	116	501	101	
Married	238 (23.5%)	21	163	54	
Divorced/living apart	52 (5.1%)	4	41	7	
Widowed	5 (0.5%)	2	2	1	
Other	1 (0.1%)				
Education					n.s.
No education	4 (0.4 %)	1	2	1	
Lower-track secondary school	25 (2.5%)	3	17	5	
Medium-track secondary school	139 (13.7%)	23	95	21	
Advanced technical college entrance qualification	140 (13.8%)	13	12	26	
Higher-track school/university entrance level	695 (68.5%)	102	85	102	
other	11 (1.1%)	0	5	6	
Highest education level					n.s.
No professional training	110 (10.8%)	17	78	15	
Apprenticeship	220 (21.7 %)	33	147	40	
Technical college	91 (9.0%)	17	58	16	
Advanced technical college	75 (7.4%)	6	51	18	
University	376 (37.1%)	46	272	58	
In training	116 (11.4%)	21	80	15	
Other	26 (2.6%)	3	21	2	
Employment Status					n.s.
Full-time	475 (46.8%)	55	331	89	
Part-time	232 (22.9%)	33	169	30	
Other^a^	306 (30.3%)	55	208	43	
Monthly income (in Euro)					n.s.
< 1,250	417 (41.1%)	68	298	51	
1,250–1,750	152 (15.0%)	25	103	24	
1,750–2,250	125 (12.3%)	20	82	23	
2,250–3,000	125 (12.3%)	12	89	24	
3,000–4,000	95 (9.4%)	9	70	16	
4,000–5,000	45 (4.4%)	3	30	12	
>5,000	55 (5.4%)	6	35	14	

*ME, Music Empathizer; B, Balanced; MS, Music Systemizer*.

a *Other included housewife/househusband, in training, unemployed, on sick leave, retired, p-values indicate whether there were significant differences between ME and MS*.

### Statistical analyses

Confirmatory Factor Analysis (CFA) was performed using R as described by Rosseel (2012). Interpretation of fit indices was conducted as recommended by Hooper et al. (2008).

Cut-off values for classifying ME, Balanced and MS were calculated according to Wakabayashi et al. (2006). Thus, for each participant, the difference score ME-MS using the unweighted raw sum scores was calculated by subtracting the mean of the MS scale from the mean of the ME scale. Thus, a high difference score can be attained either by a high ME score combined with a low MS score, or vice versa. A low difference score means that the difference between scores on the ME and MS scale is small. The greater the difference score in a positive direction, the stronger is one's ability for music empathizing, and the greater the difference score in a negative direction, the stronger is one's ability for music systemizing. The distribution (based on M and SD) of this difference score was used to determine cut-off values for ME, Balanced, and MS. Cut-off values were then determined in the following way: When a difference score was in the range of ±1 SD around the mean of the distribution of the difference score ME-MS, an individual fell into the Balanced category. Music Systemizers were characterized by a score in the range of ≤ −1 SD below the mean of the difference variable. Music Empathizers were characterized by a score in the range of ≥+1 SD above the mean of the difference variable. Thus, the discrepancy between the two dimensions of ME and MS was used to determine the cognitive style of music listening, with discrepancies in one direction (MS > ME) or the other (ME > MS) allowing for classification as either ME, MS, or Balanced.

In accordance with Wakabayashi et al. (2006), it is possible to differentiate extreme Systemizers from Systemizers within the group of Systemizers and extreme Empathizers from Empathizers within the group of Empathizers. Accordingly, extreme Music Empathizers were characterized by a score greater +2 SD above the mean, and extreme Music Systemizers were characterized by a score lower than −2 SD below the mean, respectively. Although we will report cut-off values for extreme ME and MS, this distinction will not be made when reporting differences in music preferences. Cronbach's alpha is reported as a measure of internal consistency.

In a next step, using these cut-off values, the total sample was divided *post hoc* into either ME, Balanced, or MS. Associations of ME, Balanced, and MS with sociodemographic characteristics and music preference were analyzed using SPSS. In the case of nominal data, Chi-Square (χ^2^) statistics were used. In the case of continuous data, analyses were conducted using MANOVA, and Eta Square (η^2^) is reported as a measure of effect size. Mean and standard deviation or standard error of the mean are presented where appropriate. *P* ≤ 0.05 were considered as significant.

## Results

### Confirmatory factor analysis (CFA)

To evaluate the goodness of fit of the two-factor solution of the MEMS Inventory, a CFA was conducted, χ^2^_(134)_ = 639.876, *p* ≤ 0.001. Other fit indices also revealed an adequate fit: The root mean square error of approximation was RMSEA = 0.061 (CI [0.056; 0.066], *p* ≤ 0.001), which can be interpreted as a good fit (MacCallum et al., 1996). The goodness-of-fit statistic (GFI = 0.928) can be interpreted as an acceptable fit (Hooper et al., 2008). With regard to the Comparative Fit Index (CFI = 0.872), a medium/moderate fit can be assumed, as a value of greater ≥ 0.95 is considered a good fit (Hooper et al., 2008). The same applies to the Tucker-Lewis Index (TLI = 0.854), with values greater ≥ 0.95 indicating a good fit (Hooper et al., 2008). For this two-factor model, the correlation between the factors is 0.36. Thus, although the two factors were interrelated, the correlation was only moderately positive, indicating that the two subscales do not represent the same latent variable. This two-factor solution was superior to a one-factor solution, as indicated by the χ^2^ difference test, χ^2^_(1)_ = 1019.9, *p* ≤ 0.001.

### Determination of cut-off values

The mean of the difference score ME-MS was 0.0157 ± 0.65355 in the total sample. This difference score ranged from −2.33 to 2.00 and was normally distributed (Kolmogorov-Smirnov test, *p* ≤ 0.001) with a skewness of 0.032 (*SE* = 0.077) and a kurtosis of 0.061 (*SE* = 0.153). Based on the distribution of the difference score, cut-off values for the classification as ME or MS were calculated (see Table 3).

**Table 3:** Cut-off values for categorization of MS, ME or Balanced based in difference score ME – MS.

	**Mean of difference score ME – MS ± standard deviation**	**Extreme Music Systemizer (MS ≫ ME)**	**Music Systemizer (MS > ME)**	**Balanced (MS = ME)**	**Music Empathizer (ME > MS)**	**Extreme Music Empathizer (ME ≫ MS)**
Total sample	0.0157 ± 0.65355	< −1.29	[−1.29; −0.64]	[−0.64; 0.67]	[0.67; 1.32]	>1.32
Subsample (age 18–35)	0.1054 ± 0.65898	< −1.21	[−1.21; −0.55]	[−0.55; 0.76]	[0.76; 1.42]	>1.42
Subsample (age 36–75)	−0.1405 ± 0.61427	< −1.37	[−1.37; −0.75]	[−0.75; 0.47]	[0.47; 1.09]	>1.09

*Annotations: A difference score in the range of ± 1 SD around the mean of the distribution of the difference score ME – MS leads to a classification of an individual into the Balanced category. Music Systemizers were characterized by a score in the range of ≤ −1 SD below the mean of the difference variable. Music Empathizers were characterized by a score in the range of ≥ +1 SD above the mean of the difference variable. Extreme Music Empathizers are characterized by a score greater + 2 SD above the mean, and extreme Music Systemizers were characterized by a score lower than – 2 SD below the mean, respectively*.

As the difference score ME − MS was significantly correlated with age (*r* = −0.187, *p* < 0.001), age-dependent cut-off values were calculated in a next step. Subsequent analyses showed that there was a point of inflection for the distribution of this difference score at an age of 35. Therefore, difference scores were separately calculated for the subsample 18–35 years of age ($\bar{x}$ = 0.1054 ± 0.65898) and the subsample 36–75 years of age ($\bar{x}$ = −0.1405 ± 0.61427).

### Associations between ME, balanced, MS, and music preference

Concerning sociodemographic and music-demographic associations, there were significant differences in gender distribution across ME, Balanced, and MS (χ^2^_(2)_ = 111.534, *p* ≤ 0.001), with women more often being classified as ME than MS and men more often being classified as MS than ME. Furthermore, ME were significantly younger than MS (*F*_(2, 1011)_ = 13.210, *p* ≤ 0.001, η^2^ = 0.025). There were no gender differences concerning balanced types of music listening. No differences emerged in education (e.g., graduation), highest education level (e.g., professional training), employment status, or income (all *p* > 0.067), but cognitive styles of music listening were associated with marital status (χ^2^_(8)_ = 25.731, *p* = 0.001). Visual inspection of the data shows that unmarried participants were more often ME than MS and that married participants were more often MS than ME. However, age and marital status were significantly correlated (χ^2^_(208)_ = 676.592, *p* ≤ 0.001) and the association between MEMS and marital status did not remain significant when controlling for age as covariate (*F*_(5, 1008)_ = 1.164, *p* = 0.325).

Concerning music preference ratings, cognitive styles of music listening were not associated with the overall importance of music listening for one's life, *F*_(2, 1011)_ = 1.944, *p* = 0.133, or with average amount of time spent on music listening, *F*_(2, 1011)_ = 0.390, *p* = 0.677. However, there were differences in terms of actively engaging in musical activities, *F*_(2, 2011)_ = 40.009, *p* ≤ 0.001, η^2^ = 0.073, and in the frequency of music-induced chills, *F*_(2, 1011)_ = 7.436, *p* = 0.001, η^2^ = 0.014. *Post-hoc* analyses using repeated contrasts (and thus comparing ME to MS) show that MS more often reported actively engaging in musical activities (e.g., playing an instrument) (*p* ≤ 0.001), whereas ME more often reported experiencing music-induced chills while listening to music (*p* ≤ 0.001).

**Figure 2:**
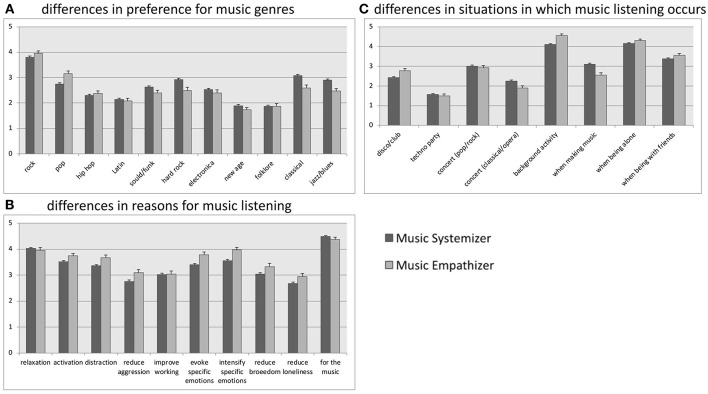
Mean preference for **(A)** each music genre, **(B)** each reason for music listening, and **(C)** situations and occasions in which music is listened to by cognitive style of music listening. Annotations: Categorization as either Music Systemizer or Music Empathizer is based on the cut-off values for the total sample; mean values are presented, separated by cognitive style of music listening (i.e., music systemizers including “extreme” music systemizers and music empathizers including “extreme” music empathizers” are presented, respectively); the “balanced” group is not displayed here; error bars represent the standard error of the mean.

Cognitive styles of music listening were associated with preferences for music genres, *F*_(22, 2004)_ = 5.653, *p* ≤ 0.001, η^2^ = 0.058: *Post-hoc* analyses using repeated contrasts show that ME and MS differed from each other in their preference for pop, rock, hip hop, hard rock, electro, classic, and jazz (Figure 2).

Reasons for music listening differed based on cognitive style of music listening, *F*_(20, 2006)_ = 4.175, *p* ≤ 0.001, η^2^ = 0.040. Repeated contrasts comparing ME to MS show that ME listen to music more regularly for the reasons of activation, distraction, reducing aggression, evoking specific emotions, intensifying specific emotions, reducing boredom, and reducing loneliness (Figure 2).

Significant differences were found in situations and occasions in which music is listened to based on cognitive style of music listening, *F*_(18, 2008)_ = 5, 166, *p* ≤ 0.001, η^2^ = 0.044. *Post hoc* contrasts comparing ME to MS show that ME visit a disco more often and listen to music as a background activity or when they are in the presence of friends. MS listen to music more often when they are making music themselves, and they reported going to classical concerts more often than ME (Figure 2).

## Discussion

### Summary of results

This study provides analyses of the psychometric properties of the German translation of the MEMS Inventory. The results demonstrate that the translated MEMS Inventory has adequate model fit while retaining the factor structure of the original English questionnaire. Based on the distribution of the difference score ME − MS, cut-off values are presented that relate the ME score to the MS score and thus allow a distinct classification as either ME, Balanced, or MS. While no differences between ME and MS emerged concerning the importance of music listening for one's life and the average duration of daily music listening, differences were found in sociodemographic variables, preferences for music genres, reasons for music listening, situations and occasions in which music is listened to, engagement with music, and the experience of music-induced chills.

### Psychometric properties of the german MEMS inventory

The two-factor structure of the MEMS Inventory was confirmed, with ME and MS representing distinct factors with adequate fit. Furthermore, the two-factor structure was superior to a one-factor model. The internal consistency of the German translation of the MEMS Inventory is comparable to that of the original English version, with coefficients for both scales that can be considered acceptable. However, the internal consistency of the ME scale is slightly higher, and the internal consistency of the MS scale slightly lower, compared to the original version. In this regard, especially item MS18 (“*I like to keep my music collection clearly ordered*, e.g., *alphabetically or by genre*.”) shows low factor loadings, although it was identified as high-loading item by Kreutz et al. (2008). On the one hand, this item might explain the lower internal consistency of the MS scale in this study. On the other hand, due to the wording of the items, the internal consistency of the MS scale might be biased toward musical sophistication. As Kreutz et al. (2008) examined participants with a high interest in music research as well as students from a music college, the wording of the MS items might, by definition, be more pertinent to this particular sample and might not be well understood in samples that are less musically sophisticated.

### Individual differences in musical experiences based on MEMS

Concerning sociodemographic variables, we replicated gender differences in MEMS as described by Kreutz et al. (2008), with women scoring higher on ME and men higher on MS, respectively. Moreover, musical engagement differed between ME and MS, as described by Kreutz et al. (2008). Furthermore, in line with Nettle (2007), we did not find differences in attraction to music as measured by importance of music listening as well as time spent listening to music. However, we did find differences in music preferences, reasons for music listening, situations in which music is listened to, engagement with music, and the experience of music-induced chills. Additionally, as assumed by Kreutz et al. (2008), reasons for engagement with music differed between ME and MS: The former seem to listen to music to achieve specific emotion regulation goals, whereas this is not the case for the latter.

Greenberg et al. (2015b) criticized that in the context of music listening, Empathizing often receives greater attention than does Systemizing. Indeed, results from empirical research suggest that Empathizing is important for understanding emotional reactions to music (Egermann and McAdams, 2013). Furthermore, as music listening in general is associated with emotion regulation (Thoma et al., 2011; Chin and Rickard, 2013), it is plausible to assume a relevant role of Empathizing in the use of music. In this regard, our results confirm that ME specifically use music to achieve these emotion regulation goals. Although Greenberg et al. (2015b) provided anecdotal evidence that people scoring high on S can also use music for emotional reasons, thus providing implications for music listening as a means of increasing empathy in Autism Spectrum Disorder, our results suggest that on a habitual level, MS do not use music for emotion regulation reasons. Rather, they prefer music of high complexity, as was also observed by Greenberg et al. (2015a).

Additionally, we were able to show that situations in which music is listened to differ based on MEMS, with ME using music in more diverse situations (e.g., various reasons for music listening, listening to music with others, music as a background activity) than MS. This again underlines the assumption that ME listen to music for various emotion regulation strategies in various situations, whereas MS use music for specific reasons of engaging in analysis of complex structures when music is in their focus of attention.

Relating our results to Sandstrom and Russo's (2013) finding that both ME and MS were correlated with strong emotional experiences while listening to music, our results suggest an association of ME with intense emotional experiences while listening to music, as measured by the habitual experience of music-induced chills.

### Potential neurophysiological mechanisms underlying musical experiences based on MEMS

This study is among the first to investigate associations between music preference/use of music and cognitive styles of music listening. It would be intriguing to examine in subsequent studies which underlying neurophysiological mechanisms account for these differences. Here, it can only be speculated which mechanisms underlie musical experiences based on MEMS.

Against the background of the neurocognitive model of music perception (Koelsch and Siebel, 2005), in which different modules of serial music perception can be distinguished, our findings might be interpreted as suggesting that ME and MS process music with differing emphases on specific modules. As we found that MS prefer music with complex structures and choose music for specific reasons that involve analyzing the structure of the music, MS might process music more elaborately with regard to feature extraction and subsequent auditory analysis. ME, however, might process music more elaborately in later stages of musical processes, when emotional analysis is conducted. At this later stage of musical processing, activity in paralimbic and limbic regions of the brain can be observed (Koelsch, 2010). Thus, there is an overlap in brain regions processing music-induced emotions and regulating stress-sensitive systems in the body, e.g., the hypothalamic-pituitary-adrenal (HPA) axis, potentially leading ME to show different patterns of physiological activity in reaction to music. Likewise, in an experimental study, Miu and Baltes (2012) manipulated empathy and showed that empathy enhanced emotions that were consistent with the valence and arousal of the music, as demonstrated by corresponding changes in physiological activity patterns with regard to heart rate and skin conductance. This mirrors our finding of ME being associated with intense emotional experiences while listening to music and a preference for reasons of music listening that are associated with emotion regulation. Especially as the general cognitive style Empathizing is associated with larger hypothalamic regions (Lai et al., 2012), it might be interesting to examine whether ME and MS vary in their ability to benefit from music for stress reduction purposes, given that the hypothalamus is part of the stress-sensitive HPA axis, which can be modulated by music listening.

### Translating these findings into practice and future directions

Understanding how MEMS shapes individual differences in musical experiences is important for the therapeutic use of music. As music listening, and in particular the reasons for music listening, is associated with emotion regulation, it is of utmost importance to understand how ME and MS can benefit from music listening. For example, in the context of music-induced analgesia, Garza Villarreal et al. (2012) were able to show that the pain-reducing effect of music differed based on the cognitive style. However, as the authors only used the general cognitive styles E-S as a framework, their results are not specific to the cognitive styles of music listening. Thus, future studies should shed further light on individual differences in musical experiences by means of MEMS and should further characterize which types of music exert beneficial effects in ME and MS. It is important to determine the extent to which the beneficial effects of music listening vary depending on cognitive styles of music listening.

## Limitations

Although the results of the study suggest adequate psychometric values, it has to be critically discussed that the categorization of MEMS is based on self-report only. In future studies, it would be intriguing to identify behavioral and neurophysiological correlates of these cognitive styles of music listening. This would enable the first-person perspective to be complemented by a third-person perspective. Furthermore, the MEMS inventory conceptualizes cognitive styles of music listening as trait variables. In addition to this trait perspective, future studies should investigate whether cognitive styles of music listening also show characteristics of state variables. In particular, ambulatory assessment studies might enable an examination of whether degrees of MEMS vary depending on characteristics associated with the music or the music listening situation.

## Conclusion

The MEMS Inventory measures the two cognitive styles of music listening ME and MS with adequate psychometric quality. Based on the distribution of the difference score ME − MS, a distinct classification of ME and MS is possible. Although ME and MS do not differ in terms of importance of music for one's life and the average amount of time spent listening to music, there are differences in reasons for music listening, situations and occasions in which music is listened to, preference for music genres, and the frequency and intensity of music-induced chills. These differences underline that ME and MS use music in different ways. From a clinical perspective, our findings suggest that ME and MS might need different musical stimulation in order to benefit from music. Future research on the psychology of music should consider MEMS as an important moderator variable. Furthermore, future research should identify behavioral and neurophysiological correlates and investigate mechanisms underlying music processing based on these different cognitive styles of music listening.

## Author contributions

All authors contributed to the conception and design of the study. AL performed statistics analysis and wrote the first draft of the manuscript. GK, MG, and UN revised the manuscript and provided input to statistical analysis and interpretation of data for the work. All authors contributed to the manuscript revision, read and approved the submitted version.

### Conflict of interest statement

The authors declare that the research was conducted in the absence of any commercial or financial relationships that could be construed as a potential conflict of interest.
