# Corrigendum: Validation of the German Version of the Music-Empathizing-Music-Systemizing (MEMS) Inventory (Short Version)

**DOI:** 10.3389/fnbeh.2018.00228

**Published:** 2018-09-28

**Authors:** Alexandra Linnemann, Gunter Kreutz, Mario Gollwitzer, Urs M. Nater

**Affiliations:** ^1^Department of Psychiatry and Psychotherapy, University Medical Center Mainz, Mainz, Germany; ^2^Department of Music, School for Linguistics and Cultural Studies, Carl von Ossietzky Universität Oldenburg, Oldenburg, Germany; ^3^Chair of Social Psychology, Department Psychology, Ludwig-Maximilians-Universität Munich, Munich, Germany; ^4^Clinical Psychology, Department of Psychology, University of Vienna, Vienna, Austria

**Keywords:** cognitive style, emotion regulation, music listening, music preference, personality, use of music

In the original article, there was an error.

In the second paragraph of the section *Associations Between ME, Balanced, MS, and Music Preference*, it is written that MS experience more music-induced chills. However, there is a spelling error and MS should be replaced by ME. A correction has been made to this paragraph which now reads:

*Post-hoc* analyses using repeated contrasts (and thus comparing ME to MS) show that MS more often reported actively engaging in musical activities (e.g., playing an instrument) (*p* ≤ 0.001), whereas ME more often reported experiencing music-induced chills while listening to music (*p* ≤ 0.001).

The authors apologize for this error and state that this does not change the scientific conclusions of the article in any way.

The original article has been updated.

## Conflict of interest statement

The authors declare that the research was conducted in the absence of any commercial or financial relationships that could be construed as a potential conflict of interest.

